# Development of a fibrin-mediated gene delivery system for the treatment of cystinosis via design of experiment

**DOI:** 10.1038/s41598-022-07750-y

**Published:** 2022-03-08

**Authors:** Valeria Graceffa

**Affiliations:** 1 Cellular Health and Toxicology Research Group (CHAT), Institute of Technology Sligo, Ash Ln, Bellanode, Sligo, Ireland; 2 Centre for Mathematical Modelling and Intelligent Systems for Health and Environment (MISHE), Institute of Technology Sligo, Ash Ln, Bellanode, Sligo, Ireland

**Keywords:** Biomedical engineering, DNA and RNA, Biotechnology, Cell delivery

## Abstract

Cystinosis is a rare disease, caused by a mutation in the gene cystinosin and characterised by the accumulation of cystine crystals. Advantages of biomaterial-mediated gene delivery include reduced safety concerns and the possibility to cure organs that are difficult to treat using systemic gene transfer methods. This study developed novel fibrin hydrogels for controlled, localised gene delivery, for the treatment of cystinosis. In the first part, fabrication parameters (i.e., DNA, thrombin, and aprotinin concentrations) were optimised, using a Design of Experiment (DOE) methodology. DOE is a statistical engineering approach to process optimisation, which increases experimental efficiency, reduces the number of experiments, takes into consideration interactions between different parameters, and allows the creation of predictive models. This study demonstrated the utility of DOE to the development of gene delivery constructs. In the second part of the study, primary fibroblasts from a patient with cystinosis were seeded on the biomaterials. Seeded cells expressed the recombinant CTNS and showed a decrease in cystine content. Furthermore, conditioned media contained functional copies of the recombinant CTNS. These were taken up by monolayer cultures of non-transfected cells. This study described a methodology to develop gene delivery constructs by using a DOE approach and ultimately provided new insights into the treatment of cystinosis.

## Introduction

Cystinosis is a rare, genetic, autosomal recessive disease, caused by a mutation in the gene cystinosin (CTNS). CTNS encodes for a lysosomal seven-transmembrane protein, that functions as an active transporter of cystine. Cystinosis is characterised by the accumulation of cystine crystals in different organs and tissues and, if inadequately treated, leads to multisystem morbidity and mortality^1^. The mainstay of treatment is the oral administration of the cystine-depleting drug cysteamine. However, this represents a life-long treatment, and it is associated with several side effects, such as stomach and bowel ulcers and bleeding. Furthermore, oral cysteamine cannot reach and effectively treat all the organs within the body^2,3^. For instance, corneal crystals can only be reduced by cysteamine eye drops. However, these are characterised by drug storage and stability problems^4,5^, and need to be administered every waking hour^6^, leading to poor compliance by patients^7^. Among new therapeutic approaches, biomaterial-mediated gene therapy potentially represents a cure for cystinosis. This approach would reduce side effects and safety concerns associated with systemic gene therapy, and it would treat difficulty accessible organs, such as the cornea^8^. Fibrin hydrogels appear good candidates, as they possess therapeutic potential alone, and are already used in the form of glue^9^, sealant^10^, or scaffolds to expand cells^11^ for clinical applications. They are prepared by mixing fibrinogen and thrombin. Fibrin self-polymerisation is initiated by the thrombin cleavage of fibrinopeptides, to produce fibrin monomers^12^. The fibrinolysis inhibitor aprotinin is often added, to increase gel stability^13^. Fibrin hydrogels have been extensively studied for tissue engineering and gene delivery purposes^14–18^. However, how the different fabrication parameters affect gene transfer is still not well understood. Available studies only compared the efficacy of two or three different parameters. For instance, a study found that hydrogels containing 25 mg/mL fibrinogen transfected murine fibroblasts more efficiently than hydrogels with 10 and 50 mg/mL of fibrinogen^15^. Similarly, hydrogels loaded with 0.5, 1 and 2 µg of DNA resulted in higher transfection efficiency, compared to gels loaded with 0.1 and 6 µg of DNA^15^. No difference in degradation rate was observed between hydrogels containing 2 U/mL or 4 U/mL of thrombin^19^. Yet, a higher rate of degradation was observed in gels with no aprotinin, compared to gels containing 50 µg/mL aprotinin^19^. However, comprehensive studies of the interaction between different parameters are missing^8^. This study aimed at developing fibrin hydrogels releasing a non-viral plasmid encoding for CTNS, which can potentially be used for the treatment of cystinosis. Optimal fabrication parameters were identified using a design of experiment (DOE) approach. Specifically, variables investigated were DNA, thrombin, and aprotinin concentrations. DOE enabled to determine factors affecting transfection efficiency, cellular viability, biomaterial stability, and their potential interaction. Primary fibroblasts from a patient with cystinosis were then seeded on the biomaterials: cellular transfection, accumulation of cystine, and secretion of the recombinant CTNS were finally evaluated.

## Materials and methods

### Materials

Unless otherwise specified, cell culture reagents were bought from Sigma-Aldrich Ireland Ltd. The expression vector pCMV6-AC-GFP and the plasmid pCMV6-AC-CTNS-GFP (containing the transcript variant 2 (NM_004937) of the human CTNS, with a C-terminal TurboGFP tag, tGFP) were bought from Origene and expanded in NEB 5-alpha Competent E. coli cells (New England Biolabs). Plasmids were isolated using the GeneJET Plasmid Maxiprep Kit (ThermoFisher Scientific) and their identity was confirmed through a 0.5% agarose electrophoresis, before and after digestion with EcoRI-HF (New England Biolabs).

### Cell culture

**Figure 1:**
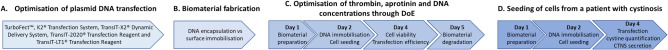
Schematic representation of the experimental process. Transfection efficiency of the reagents TurboFect, K2 Transfection System, TransIT-X2 Dynamic Delivery System, TransIT-2020 Transfection Reagent and TransIT-LT1 Transfection Reagent was initially evaluated. (**A**) DNA encapsulation and surface immobilisation were then compared. (**B**) Thrombin, aprotinin and DNA concentrations were then optimised by DOE, as shown in the timeline: biomaterials were left to polymerise overnight; the day after, DNA was surface immobilised and HDFs were seeded. Cell viability and transfection efficiency were evaluated after two days, whereas biomaterial degradation was assessed after three days. (**C**) HDFs from a patient affected by cystinosis were finally seeded on the biomaterials. Cellular transfection, intracellular cystine levels and release of the recombinant CTNS into the culture media were assessed after two days after cell seeding (**D**).

Human primary dermal fibroblasts (HDFs) from a 33 years old, female donor (Brennan & Co) and human primary dermal fibroblasts from a male cystinotic donor of 24 years old, (Coriell Institute for Medical Research) were cultured in Dulbecco’s modified Eagle’s medium high glucose DMEM-HG, without phenol red, supplemented with 10% HyClone Calf Serum (Fisher Scientific) and 1% penicillin–streptomycin solution. Cells at passages 5 to 8 were used. The experimental design and timeline are shown in Fig. 1.

### Comparison of transfection efficiency between commercial transfection reagents

Transfection efficiency of the TurboFect (ThermoFisher Scientific), the K2 Transfection System (Biontex), the TransIT-X2 Dynamic Delivery System (Mirus Bio), TransIT-2020 Transfection Reagent (Mirus Bio) and the TransIT-LT1 Transfection Reagent (Mirus Bio) was evaluated, using a reverse transfection protocol. Briefly, pCMV6-AC-CTNS-GFP was diluted in DMEM-HG, supplemented with 1% penicillin–streptomycin. Transfection reagents were then added (DNA: reagent ratios 1:2, 1:3, 1:4, or 1:5) and incubated for 20 min at room temperature to allow the formation of the complexes. Samples were then transferred to empty 48 well plates. Healthy HDFs were finally seeded in the multiwell plates with the DNA complexes, at a density of 50.000 cells/cm^2^. Transfection efficiency was evaluated after 2 days, as indicated in “Evaluation of transfection efficiency” section. The experiment was performed in triplicate and the fluorescence intensity of six images per group was quantified.

### Biomaterial preparation

Fibrinogen type I-S from bovine plasma and thrombin from bovine plasma were dissolved in NaCl 0.9% at a concentration of 3 mg/mL and at concentrations of 0.1, 0.8, 1.8, 2.8, and 3.5 U/mL, respectively. Aprotinin from bovine lung was dissolved in CaCl_2_ 200 mM at concentrations of 40, 100, 160, 200 KIU/mL. Hydrogels for the seeding of cystinosis cells were prepared with 1 µg DNA, 1.8 U/mL thrombin and with no aprotinin. After sterile filtration (0.22 µm), 50 µL of each solution was transferred to a well of a 48 multiwell plate. Gels were allowed to polymerise overnight at 37° C. K2 lipoplexes (0, 0.2, 0.5, 0.8 and 1 µg of pCMV6-AC-CTNS-GFP at a DNA: K2 ratio 1:3) were diluted with DMEM-HG to a total volume of 200 µL and then added to the polymerised gels. After one hour of incubation at 37° C, gels were washed with Cytiva HyClone Phosphate Buffered Saline (PBS) (FisherScientific). Primary fibroblasts were finally seeded on top of the gels, at a density of 50,000 cells/cm^2^.

### Comparison between DNA surface immobilisation and encapsulation

To compare immobilisation and encapsulation, pCMV6-AC-CTNS-GFP was labelled with the SYBR Green staining reagent (PanReac AppliChem), and then incubated with K2 reagent. K2 polyplexes were then either encapsulated (50 µL of complexes were mixed with 50 µL of fibrinogen, 50 µL of aprotinin and 50 µL of thrombin solution and let polymerise) or surface immobilised (gels were allowed to polymerise and then incubated with DNA complexes). Fluorescence pictures were taken using an ECLIPSE Ts2 inverted fluorescence microscope (Nikon). This experiment was performed in triplicate and ten images per group were taken.

### Quantification of DNA released

Biomaterials loaded with pCMV6-AC-CTNS-GFP polyplexes were washed and incubated in Cytiva HyClone PBS (ThermoFisher Scientific), at 37 °C. PBS was taken at different time intervals and replaced with an equal volume of fresh buffer. DNA released in the buffer was quantified using a DS-11 Spectrophotometer (DeNovix Inc). Table 2 shows the amount of DNA (*µ*g) released, whereas Fig. 5 presents results as a percentage of the total DNA loaded.

### Cellular viability

Cellular viability was assessed using the Resazurin Assay Kit (Abcam), following manufacturers’ instructions. Briefly, cells (50,000 cells/ cm^2^) were incubated with Resazurin reagent diluted 1:20 in DMEM-HG (150 µL/ cm^2^), for 45 min at 37 °C. Oxidation of resazurin was quantified measuring the fluorescence (530 nm excitation and 570 nm emission), with a FLUOstar OPTIMA microplate reader (BMG Biotech). Results are expressed as a percentage of resazurin reduced of control (monolayer cultures of non-transfected cells).

### Evaluation of transfection efficiency

Transfection efficiency was evaluated two days after cell seeding (50,000 cells/ cm^2^). Fluorescence pictures were taken using an ECLIPSE Ts2 inverted fluorescence microscope (Nikon). Mean fluorescence intensity of at least five 20 × microscope fields was quantified using ImageJ software (National Institutes of Health, NIH). Data are shown as mean ± standard deviation.

### Assessment of biomaterial degradation

Biomaterial degradation was evaluated three days after cell seeding (50,000 cells/ cm^2^). Images of the pCMV6-AC-CTNS-GFP loaded hydrogels were converted to an 8-bit, using ImageJ. Biomaterials were then cropped. The mean gray area was quantified and presented as a percentage of freshly made gels.

### Quantification of cystine

Fibroblasts from a cystinosis patient were seeded on hydrogels loaded with pCMV6-AC-CTNS-GFP and pCMV6-AC -GFP, at a density of 50,000 cells/cm^2^. After two days, hydrogels were hydrolysed by trypsinisation. Cells were then dissolved in 1 N HCl and lysed by freeze–thaw. Lysates were analysed through UV–VIS spectroscopy, and cystine was quantified using a L-Cystine standard curve. UV–VIS calibration curve and spectroscopy of cell lysates are shown in Supplementary Figure S1.

### Immunoprecipitation and SDS-PAGE

Fibroblasts from a cystinosis patient were seeded on fibrin hydrogels loaded with pCMV6-AC-CTNS-GFP, at a density of 50,000 cells/cm^2^. Culture media was harvested after two days and subjected to ultrafiltration (Amicon Ultra, MWCO 100 kDa). To facilitate antibody access to the tagged protein, concentrated media was then treated with a lysis buffer, (10 mM Tris–HCl pH 7.5, 150 mM NaCl, 0.5 mM EDTA, 0.5% TritonX-100), supplemented with *α*-Toluenesulfonyl fluoride 1 mM (ThermoFisher Scientific) and Fisher Bioreagents Protease Inhibitor Cocktail V, EDTA Free (ThermoFisher Scientific). Immunoprecipitation was performed using the TurboGFP-Trap Agarose (Chromotek), following manufacturers’ instructions. Culture media from non-transfected cells was used as a negative control. After the immunoprecipitation, the recombinant CTNS was eluted with 2X SDS-PAGE samples buffer and separated in a 4–12% Bis–Tris gradient gel. Gels were finally stained using the Pierce Silver Stain Kit (ThermoFisher Scientific).

### Preparation of conditioned media

Fibroblasts from a patient with cystinosis were seeded on hydrogels loaded with the pCMV6-AC-CTNS-GFP, at a density of 50,000 cells/cm^2^. Culture media was harvested after two days, centrifuged at 1,200 rpm for 5 min to remove cellular debris, diluted 2:3 with fresh culture media, and then added to confluent monolayer cultures of non-transfected cystinotic fibroblasts. Conditioned medium from non-transfected cells was used as a negative control. The days after, cells were washed with PBS and analysed at an ECLIPSE Ts2 inverted fluorescence microscope (Nikon). In some experiments (Fig. 9E), cells were incubated with Hoechst 33,258 15 µg/mL in PBS for 15 min at 37 °C, before fluorescence microscopy.

### Statistics and design of experiment (DOE)

Minitab software was used for statistical analysis. Data are presented as mean ± standard deviation. Each experiment contained at least three replicates (the number of replicates N is indicated in the figure legends). Unless otherwise specified, all experiments were performed twice, to confirm the reproducibility. Design of experiment (DOE) was performed using Minitab program. A surface response (axial point, 0, 20, 50, 80, 100 µL of DNA complexes, 0.1, 0.8, 1.8, 2.8 or 3.5 U/mL of thrombin, 40, 100, 160, or 200 KIU/mL of aprotinin) was used to determine factors affecting cellular transfection, viability, and biomaterial stability and to draw the contour plots.

## Results

### Optimisation of transfection system and DNA conjugation strategy

**Figure 2:**
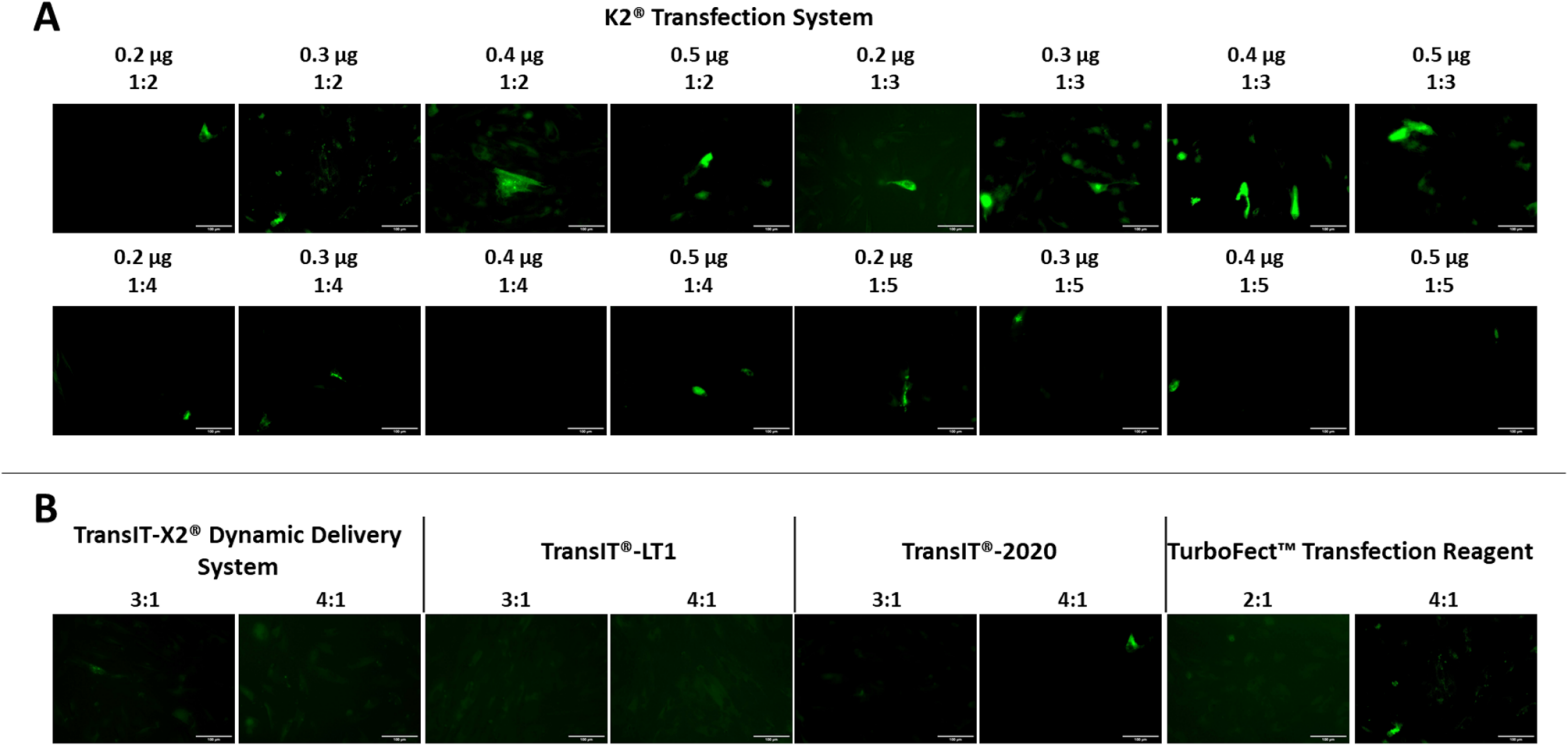
Comparison of transfection efficiency between commercial transfection reagents. HDFs were transfected using the K2 Transfection System (**A**), the TransIT-X2 Dynamic Delivery System, the TransIT-2020 Transfection Reagent, the TransIT-LT1 Transfection Reagent and TurboFect (**B**) Fluorescence images were taken after 2 days. N = 3, with six images per group.

**Figure 3:**
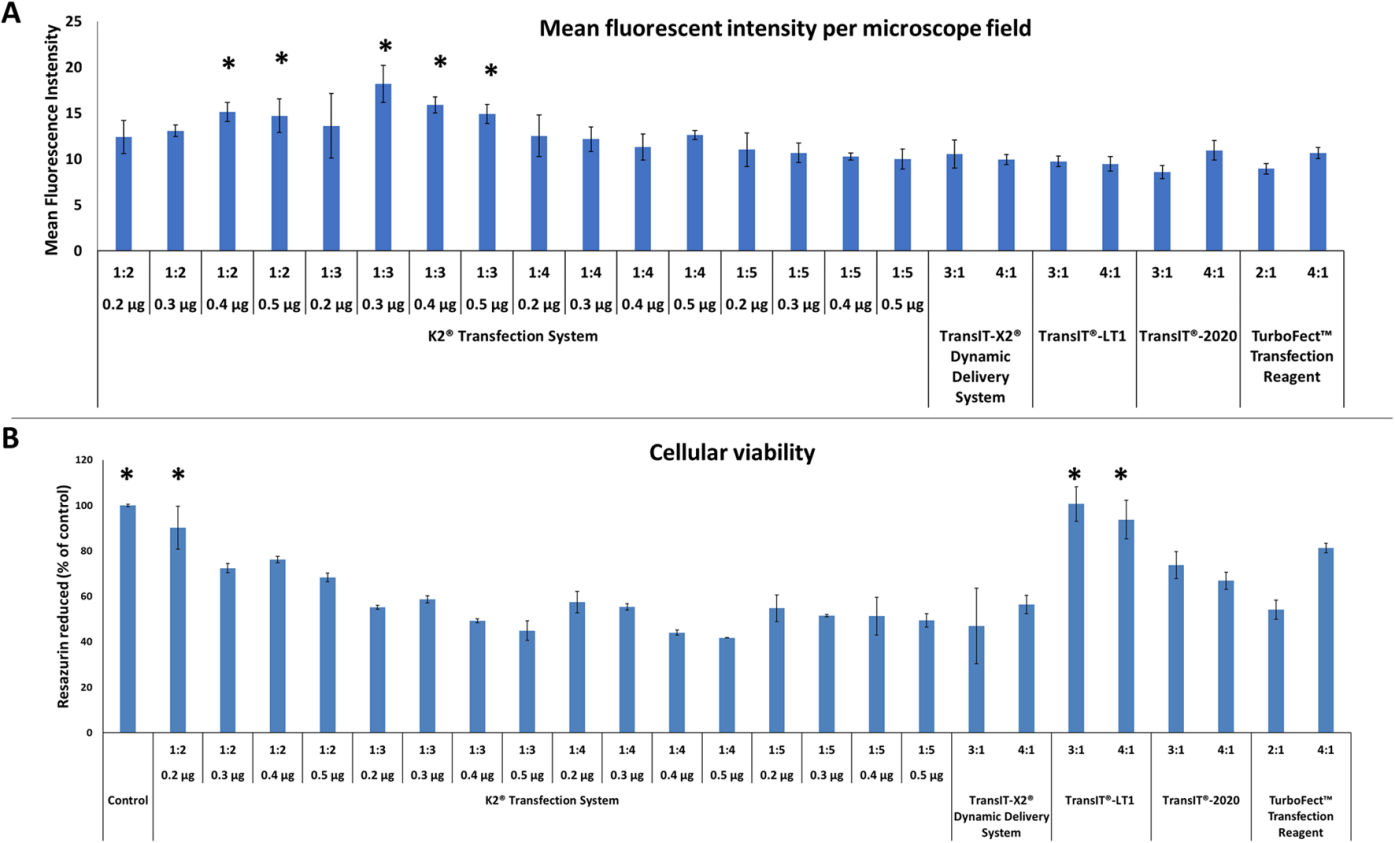
Comparison of transfection efficiency between commercial transfection reagents. HDFs were transfected using the K2 Transfection System, the TransIT-X2 Dynamic Delivery System, the TransIT-2020 Transfection Reagent, the TransIT-LT1 Transfection Reagent and TurboFect and analysed after two days. Quantification of mean fluorescence intensity per microscope field is shown in (**A**) and cellular viability is shown in (**B**). ANOVA statistical test. *p ≤ 0.05. N = 3.

**Figure 4:**
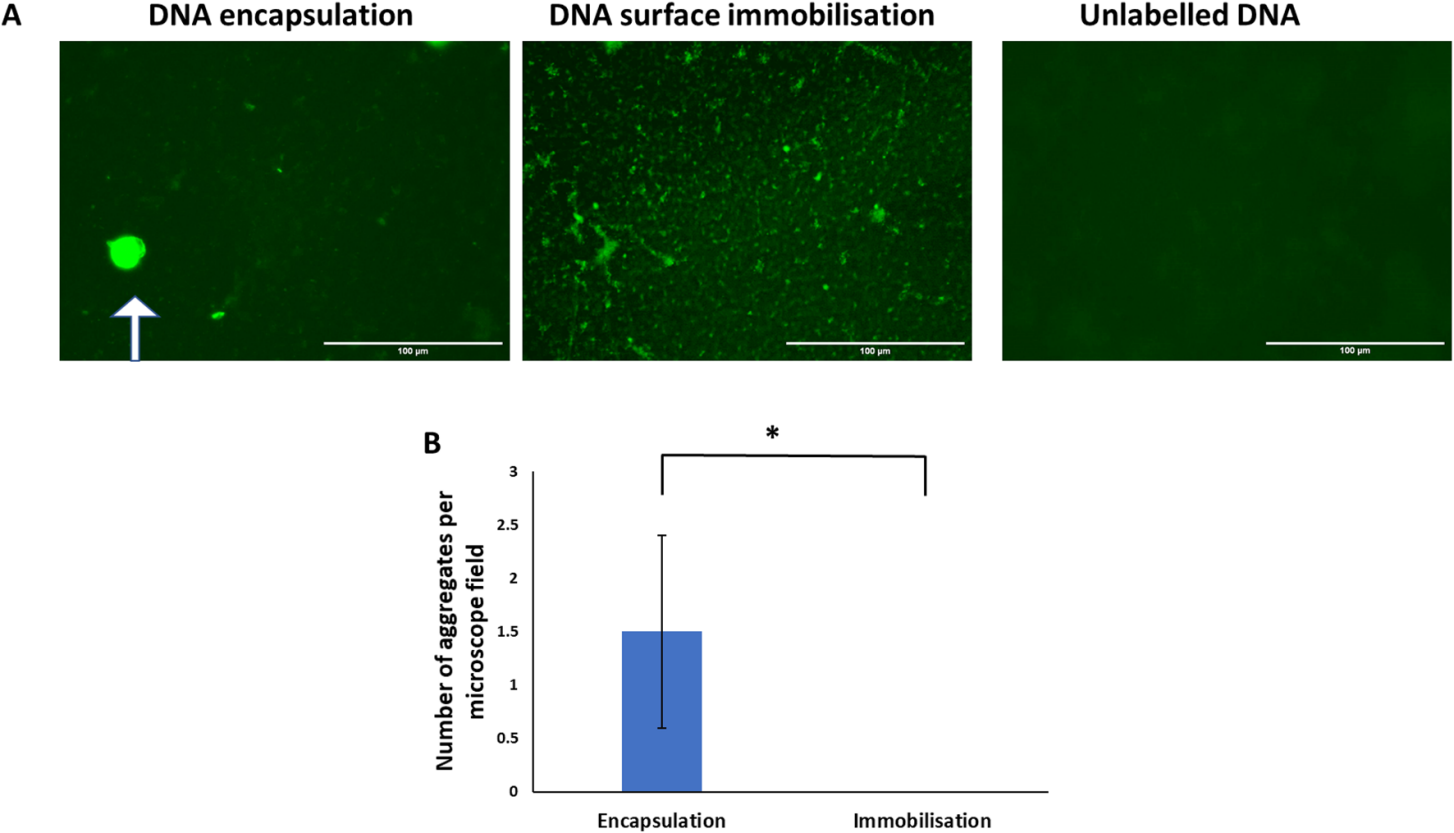
Comparison between DNA encapsulation and surface immobilisation. SYBR-Green labelled DNA was complexed with K2 reagent and either encapsulated (polyplexes were mixed with fibrinogen, thrombin and aprotinin and let polymerise), or surface immobilised (fibrin hydrogels were polymerised and then incubated with DNA polyplexes). Representative images are shown in (**A**) and quantification of number of aggregates per microscope field in (**B**). Encapsulation resulted in the formation of large DNA aggregates, as highlighted by the white arrow. T-test statistics. *p ≤ 0.05. N = 3, with ten images per group.

As shown in the sequence diagram in Fig. 1, in a first step, the transfection efficiency of the reagents TurboFect, K2 Transfection System, TransIT-X2 Dynamic Delivery System, TransIT-2020 Transfection Reagent, and TransIT-LT1 Transfection Reagent was evaluated. As shown in Fig. 2A–B, K2 led to the highest transfection efficiency. Particularly, 0.3 µg of DNA in a DNA: K2 ratio 1:3 significantly differed from all the other conditions tested, whereas DNA: K2 ratio 1:3, 0.4 and 0.5 µg and DNA: K2 ratio 1:2, 0.4 and 0.5 µg significantly differed from TransIT-X2 Dynamic Delivery System, TransIT-LT1, TransIT-2020 and TurboFect Transfection Reagent (Fig. 3A). Cellular viability was slightly reduced in all groups compared to non-transfected cells, except for the TransIT-LT1 system (Fig. 3B). In all subsequent experiments, the K2 reagent, at a 1:3 DNA: reagent ratio was used. DNA-containing scaffolds are typically fabricated through either encapsulation or immobilisation^8^. Thus—as indicated in the sequence diagram in Fig. 1- in the second step, DNA encapsulation and surface immobilisation were compared. As shown in Fig. 4A–B, the former approach resulted in the formation of large aggregates. When surface immobilised, DNA complexes appeared instead more uniformly distributed within the gels, with no visible aggregates. Based on this result, all subsequent hydrogels were prepared via DNA immobilisation.

### DOE allowed the identification of variables affecting transfection efficiency, cellular viability, and biomaterial degradation rate

**Figure 5:**
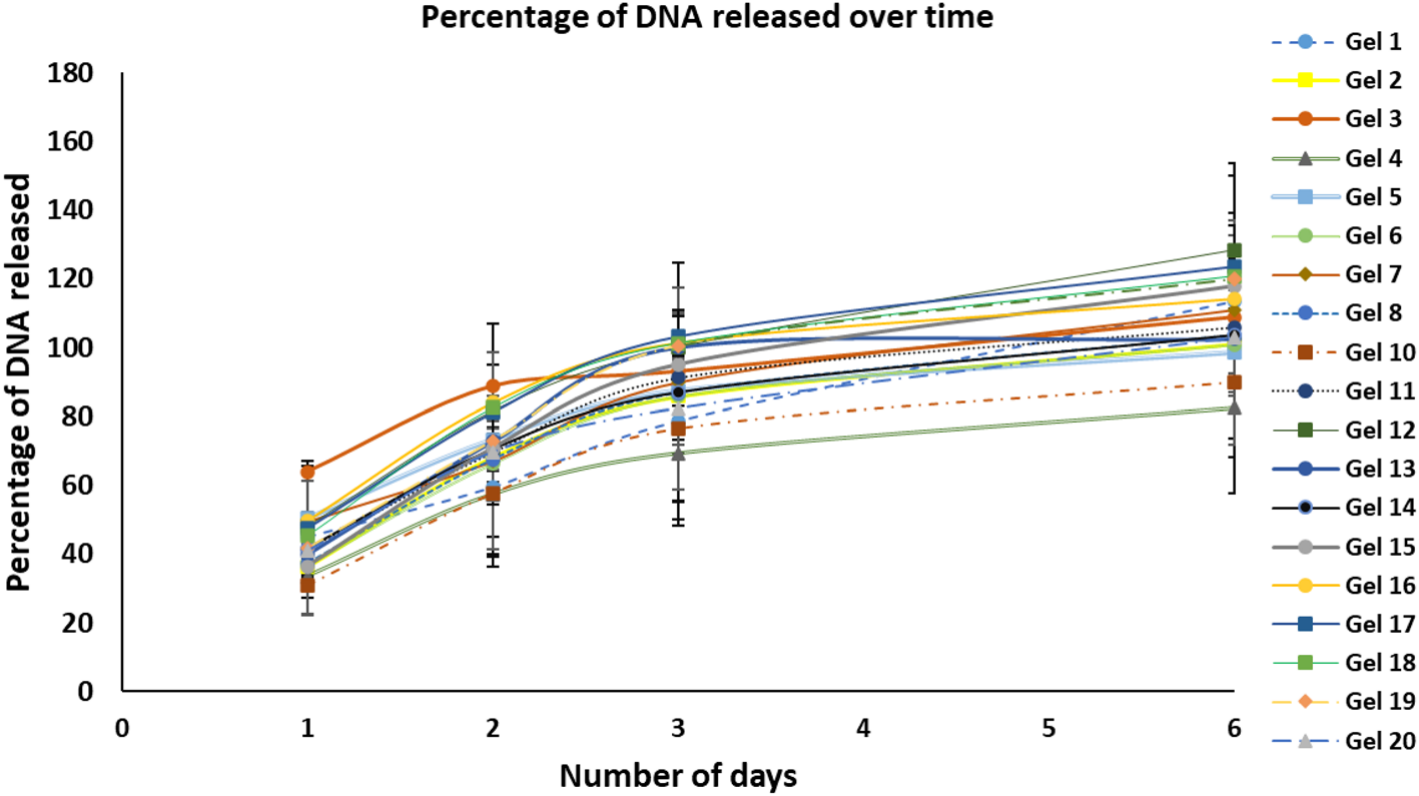
Percentage of DNA released over time. Hydrogels were prepared using different concentrations of thrombin, aprotinin and DNA (concentrations are indicated in Table 2). The percentage of DNA released was measured at different timepoints. DNA was completely released after 6 days, and a similar pattern was observed for all groups. N = 3.

As indicated in the sequence diagram in Fig. 1, DOE was then used to identify variables affecting transfection efficiency, cellular viability, and biomaterial degradation. Literature review and preliminary investigations were used to select fibrinogen concentration, and the initial variables, with the appropriate levels. Variables tested can be found in Table 1. To confirm immobilisation of polyplexes and to study the kinetics of release, biomaterials were incubated with PBS. DNA released into the saline solution was quantified, as shown in Table 1. Figure 5 indicates DNA released, as a percentage of the total amount initially loaded. DNA was completely released in less than 6 days, and a similar pattern was observed for all groups.

**Table 1:** Quantification of DNA released over time from fibrin hydrogels. Fibrin hydrogels prepared using different concentrations of DNA, aprotinin and thrombin, were incubated in PBS. DNA released was quantified at different timepoints. N = 3.

Gel #	Variables			DNA released (µg)			
	DNA µg	Thrombin (U/mL)	Aprotinin (KIU/mL)	Day 1	Day 2	Day 3	Day 6
1	0.2	0.8	40.5	0.096 ± 0.005	0.128 ± 0.03	0.165 ± 0.032	0.233 ± 0.042
2	0.8	0.8	40.5	0.296 ± 0.01	0.555 ± 0.02	0.694 ± 0.037	0.815 ± 0.049
3	0.2	2.8	40.5	0.128 ± 0.007	0.177 ± 0.008	0.186 ± 0.015	0.218 ± 0.019
4	0.8	2.8	40.5	0.274 ± 0.05	0.469 ± 0.1	0.563 ± 0.11	0.667 ± 0.114
5	0.2	0.8	160	0.107 ± 0.033	0.156 0.0667	0.182 ± 0.075	0.204 ± 0.081
6	0.8	0.8	160	0.300 ± 0.027	0.540 ± 0.054	0.695 ± 0.065	0.814 ± 0.081
7	0.2	2.8	160	0.105 ± 0.03	0.143 ± 0.056	0.187 ± 0.07	0.228 ± 0.078
8	0.8	2.8	160	0.304 ± 0.029	0.549 ± 0.059	0.702 ± 0.077	0.835 ± 0.088
9	0	1.8	100	0.006 ± 0.003	0.007 ± 0.006	0.007 ± 0.018	0.007 ± 0.03
10	1	1.8	100	0.315 ± 0.082	0.586 ± 0.165	0.774 ± 0.177	0.907 ± 0.183
11	0.5	0.1	100	0.214 ± 0.037	0.357 ± 0.075	0.464 ± 0.089	0.536 ± 0.099
12	0.5	3.5	100	0.247 ± 0.054	0.417 ± 0.108	0.508 ± 0.121	0.649 ± 0.137
13	0.5	1.8	0	0.205 ± 0.005	0.373 ± 0.009	0.508 ± 0.012	0.517 ± 0.025
14	0.5	1.8	200	0.216 ± 0.016	0.362 ± 0.031	0.442 ± 0.053	0.525 ± 0.065
15	0.5	1.8	100	0.188 ± 0.032	0.363 ± 0.052	0.483 ± 0.078	0.596 ± 0.088
16	0.5	1.8	100	0.255 ± 0.044	0.429 ± 0.088	0.515 ± 0.102	0.5782 ± 0.12
17	0.5	1.8	100	0.244 ± 0.014	0.415 ± 0.024	0.524 ± 0.033	0.625 ± 0.044
18	0.5	1.8	100	0.233 ± 0.013	0.421 ± 0.026	0.514 ± 0.044	0.61 ± 0.05
19	0.5	1.8	100	0.215 ± 0.022	0.374 ± 0.045	0.51 ± 0.053	0.606 ± 0.078
20	0.5	1.8	100	0.210 ± 0.013	0.358 ± 0.026	0.42 ± 0.0662	0.522 ± 0.085

**Figure 6:**
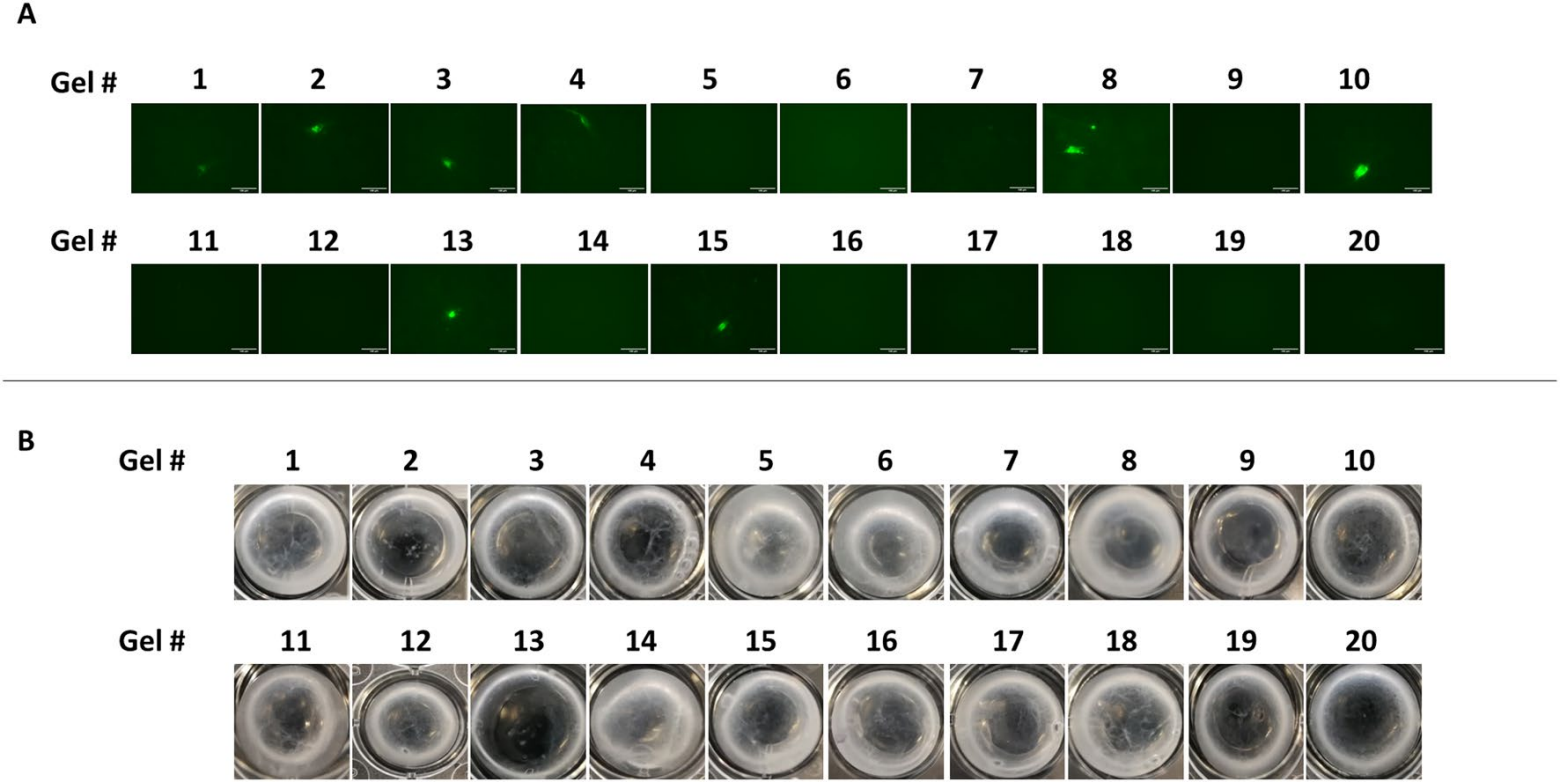
Transfection efficiency and degradation rate of fibrin hydrogels. Healthy fibroblasts were seeded on hydrogels prepared using different concentrations of thrombin, aprotinin and DNA (concentrations are indicated in Table 2). Fluorescence images were taken after two days (**A**), whereas brightfield pictures of the biomaterials were taken after three days (**B**). N = 3.

**Figure 7:**
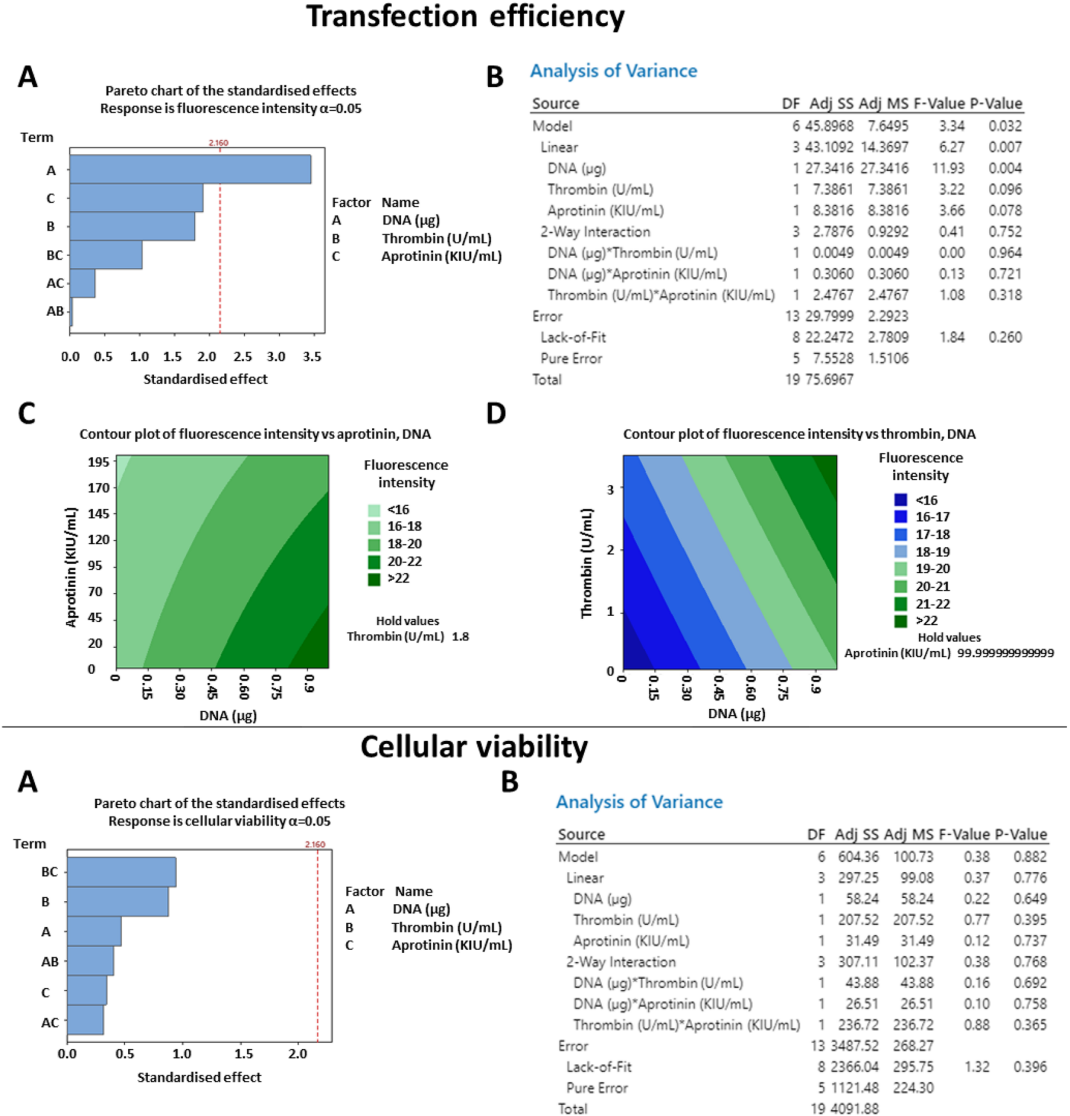
Characterisation of variables affecting transfection efficiency and cellular viability, through design of experiment. A response surface design was used to determine whether aprotinin, thrombin or DNA modulated transfection efficiency (**A**–**D**) and cellular viability (**E**–**F**). As shown in the Pareto chart (**A**) and in the ANOVA table (**B**), DNA concentration was the only factor significantly affecting transfection efficiency. Surface contour plots show how the mean fluorescence intensity per microscope field varied at different concentrations of DNA and aprotinin (**C**) or at different concentrations of DNA and thrombin (**D**). None of the variable tested significantly affected cellular variability (**E**), which did not significantly vary between the groups (**F**). N = 3. In A-D, five images were taken per group and the average values were analysed using Minitab.

To evaluate the transfection efficiency, healthy fibroblasts were seeded on the hydrogels and analysed by fluorescence microscopy two days after. Representative images are shown in Fig. 6A and mean fluorescence intensity per microscope field is reported in Table 2. As shown in the Pareto chart in Fig. 7A and in the corresponding ANOVA table in Fig. 7B, DNA was the main factor linearly affecting transfection efficiency. The highest levels of fluorescence intensity were achieved for 1 µg of DNA (gel #10 in Table 2). A trend toward an increase in transfection at high thrombin was observed. This may indicate a possible correlation between surface stiffness and DNA uptake. Furthermore, increasing the concentration of thrombin may alter the surface charge of the scaffold, and it may result in a more densely cross-linked network, and low porosity^20^. This could have also influenced cellular behaviour.

**Table 2:** Characterisation of fibrin gels through design of experiment. A response surface design was used to determine whether aprotinin, thrombin or DNA affected transfection efficiency, cellular viability and biomaterial stability. Results are representative of three independent experiments, with N = 3.

Gel #	Variables			Responses		
	DNA (µg)	Thrombin (U/mL)	Aprotinin (KIU/mL)	Mean Fluorescence Intensity	Cellular viability	% of remaining biomaterial at day 3
1	0.2	0.8	40.5	18.19 ± 2.47	77.86 ± 1.4	49.98 ± 22.02
2	0.8	0.8	40.5	21.95 ± 1.04	80.1 ± 0.92	52.6 ± 6.782
3	0.2	2.8	40.5	19.83 ± 2.06	60.03 ± 7.54	62.96 ± 16.93
4	0.8	2.8	40.5	21.42 ± 2.37	73.54 ± 1.67	40.8 ± 6.98
5	0.2	0.8	160	16.9 ± 2.938	66.56 ± 2.6	80.91 ± 2.6
6	0.8	0.8	160	19.61 ± 1.81	63.42 ± 8.4	76.84 ± 2.4
7	0.2	2.8	160	18.5 ± 1.54	72.39 ± 0.98	72.5 ± 0.9
8	0.8	2.8	160	21.58 ± 4.57	76.72 ± 12.28	71 ± 7.5
9	0	1.8	100	16.7 ± 0.293	70.35 ± 13.79	54.56 ± 1.84
10	1	1.8	100	22.76 ± 0.72	73.15 ± 3.5	49.43 ± 2.87
11	0.5	0.1	100	16.4 ± 1.77	67.82 ± 13.75	57.25 ± 5.73
12	0.5	3.5	100	17.4 ± 1.27	102.6 ± 17.26	70.4 ± 10.9
13	0.5	1.8	0	20.37 ± 3.91	87.75 ± 3.93	3.986 ± 1.34
14	0.5	1.8	200	18.06 ± 1.59	85.51 ± 24.17	84.72 ± 14.11
15	0.5	1.8	100	20.58 ± 2.4	74.55 ± 5.58	66.12 ± 0.93
16	0.5	1.8	100	18.11 ± 1.51	65.38 ± 5.435	64.54 ± 0.88
17	0.5	1.8	100	18.82 ± 0.51	67.89 ± 17.47	64.52 ± 3.58
18	0.5	1.8	100	16.83 ± 3.09	66.38 ± 17.38	67.105 ± 4.36
19	0.5	1.8	100	18.45 ± 0.9	86.2 ± 11.4	51.7 ± 14.78
20	0.5	1.8	100	18.09 ± 1.13	97.51 ± 0.12	47.59 ± 9.85

On the other hand, albeit non significantly, lower aprotinin concentration was associated with higher transfection efficiency. No interactions between any of the variables was observed. Contour plots in Fig. 7C and D show the prediction of fluorescence intensity at variable concentrations of DNA, thrombin, and aprotinin.

As shown in Table 2, cellular viability was similar among the groups and was not significantly affected by any of the variables tested (Fig. 7E–F). This indicated good biocompatibility of the biomaterial. Based on these results, all subsequent gels were loaded with 1 µg of DNA, as this condition led to the highest transfection efficiency, without compromising cellular viability.

**Figure 8:**
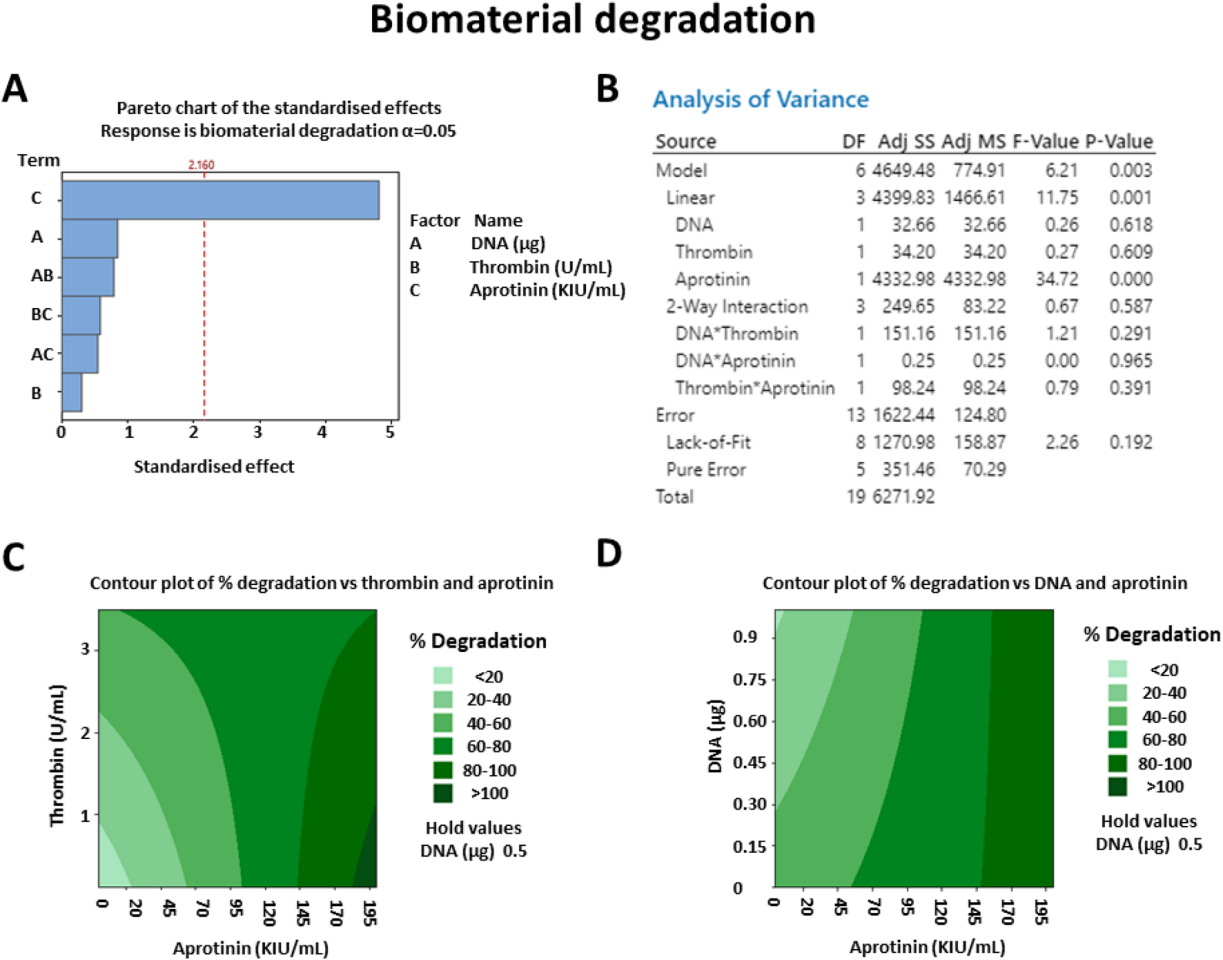
Characterisation of variables affecting biomaterial degradation through design of experiment. A response surface design was used to determine whether aprotinin, thrombin or DNA modulated hydrogels stability. As shown in the Pareto chart (**A**) and in the ANOVA table (**B**), aprotinin was the only factor significantly affecting biomaterial degradation. Surface contour plots show how biomaterial degradation varied at different concentrations of aprotinin and thrombin (**C**) or at different concentrations of aprotinin and DNA (**D**). N = 3.

If further developed, the hydrogels could be applied to the cornea, and used for the treatment of ocular cystinosis. Natural polymer-based gels are used to fill corneal defects^21,22^ or to deliver cells^11^ for ocular tissue engineering purposes. After implantation, the grafted area is covered with surgical dressing. Gels are generally required to degrade in 2–4 days, for safety concerns and patients’ comfort, and to reduce the risk of infection^23,24^. DOE was thereby used to identify parameters affecting biomaterial stability. Brightfield images of the hydrogels after three days in culture are shown in Fig. 6B, whereas the estimated percentage of degradation is indicated in Table 2. The concentration of aprotinin was the main factor determining stability, as shown by the Pareto chart in Fig. 8A and by the corresponding ANOVA table in Fig. 8B. Gels without aprotinin (gel # 13 in Table 2) were dissolved in three days, whereas gels with a high concentration of aprotinin (gel #14 in Table 2) remained undegraded. Figure 8C–D shows a prediction of biomaterial remaining at day 3, at different thrombin, DNA, and aprotinin concentrations. Based on this result, all subsequent gels were prepared without aprotinin.

### Fibrin hydrogels allowed diseased cells to synthesize and secrete the recombinant CTNS

**Figure 9:**
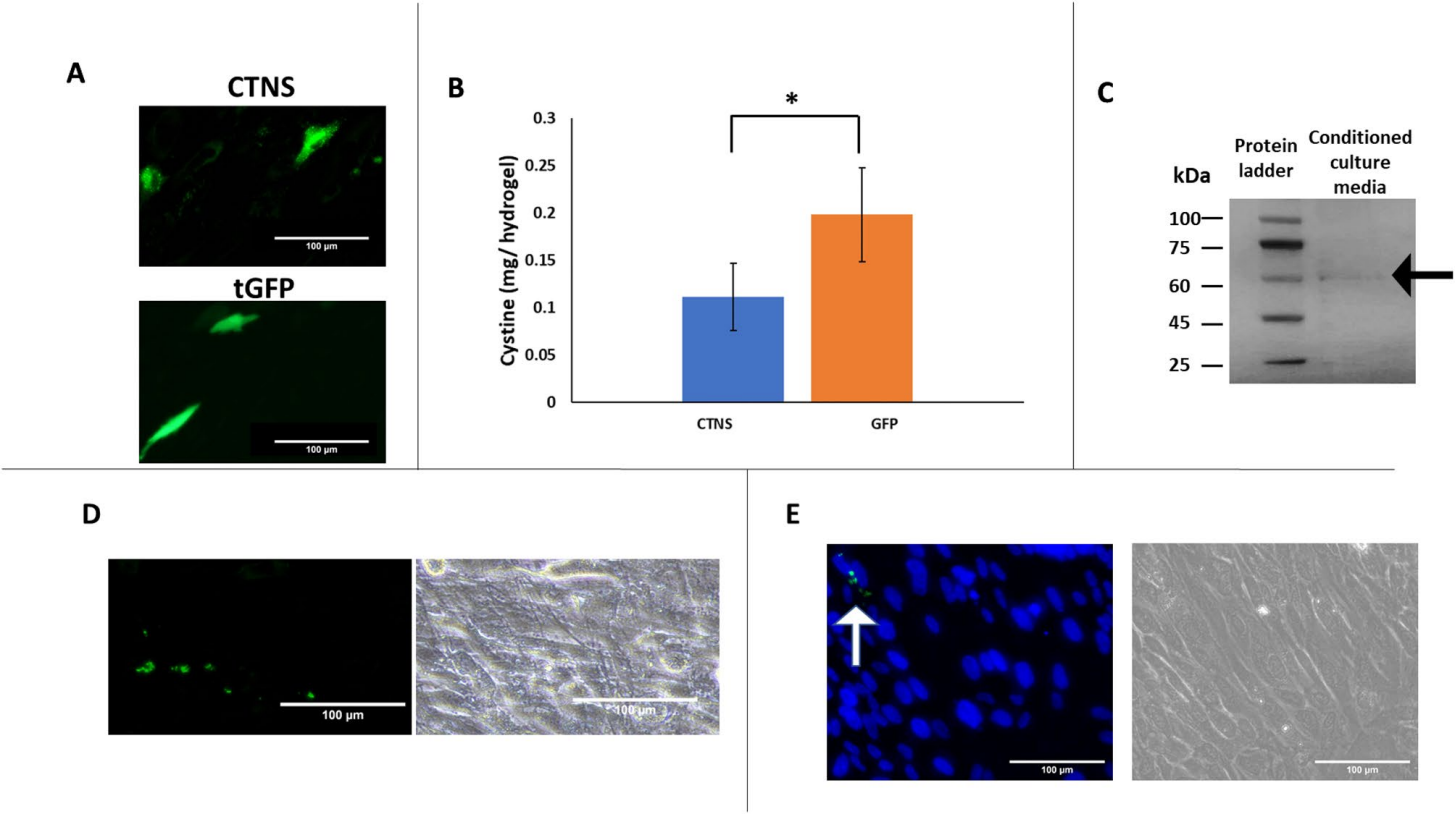
Seeding of cells from a patient with cystinosis on fibrin hydrogels. Cells from a patient with cystinosis were seeded on hydrogels, loaded with either CTNS-encoding plasmid (top), or tGFP empty vector as a control (bottom) (**A**). Two days after, cystine levels were significantly reduced in CTNS group. T-test statistics. * *p* ≤ 0.05 N = 4 (**B**). TurboGFP-immunoprecipitation of culture media was performed, and secreted CTNS was detected by SDS-PAGE N = 3 (**C**). Non-transfected cells were cultured with conditioned media from the hydrogels. The day after, fluorescence images (on the left), with their corresponding brightfield images (on the right) were taken. Sparse green, fluorescent dots were visible N = 3 (**D**). To confirm intracellular localisation, in some experiments, nuclei of live cells were counterstained with Hoechst 33,258. Fluorescence images (on the left), with their corresponding brightfield images (on the right) were taken. The white arrow indicates the green, fluorescent dots N = 3 (**E**).

Primary fibroblasts from a patient with cystinosis were finally seeded on hydrogels prepared using parameters optimised in “Optimisation of transfection system and DNA conjugation strategy” and “DOE allowed the identification of variables affecting transfection efficiency, cellular viability, and biomaterial degradation rate” sections. Hydrogels loaded with the empty vector pCMV6-AC-GFP were used as control (group tGFP in Fig. 9). As shown in Fig. 9A, two days after cell seeding, many tGFP-expressing cells were observed. Particularly, for the empty plasmids, the fluorescent signal appeared uniform within the cytoplasm, whereas for the CTNS-encoding plasmids, a dot-like pattern was observed, indicating that the tagged protein localised to lysosomes. As shown in Fig. 9B, the amount of cystine was significantly reduced for CTNS-encoding plasmids. Lysosomal proteins are generally secreted to the extracellular space, following traditional exocytic and endocytic pathways^1^. To verify whether the recombinant CTNS was secreted, culture media was analysed through TurboGFP immunoprecipitation, followed by SDS-PAGE. As shown in Fig. 9C, a band of ∼ 60 kDa corresponding to the TurboGFP-tagged recombinant CTNS was detected in the conditioned media from cell-seeded biomaterials. To verify whether the secreted CTNS could also be endocytosed, conditioned media was used to cultivate non-transfected cystinosis fibroblasts. Some sparse green fluorescent dots were observed the day after conditioning (Fig. 9D). To confirm intracellular localisation, in some experiments, nuclei of live cells were counterstained with Hoechst 33,258 (Fig. 9E). These results indicated that the recombinant CTNS followed traditional exocytic and endocytic pathways.

## Discussion

Gene therapy offers new hopes against many incurable disorders, and potentially represents a final cure for many diseases. Among potential approaches, advantages of biomaterial-mediated gene delivery include reduced side effects/ safety concerns and the possibility to cure organs that are difficult to treat with systemic delivery methods. There currently is a research group assessing ex vivo gene therapy^25,26^, but no study ever developed a biomaterial-guided gene delivery system for the treatment of cystinosis. This approach would be particularly useful in the treatment of ocular symptomatology, as this is extremely difficult to cure. Novel treatments under investigation for ocular cystinosis include new viscous cysteamine hydrochloride eye drops^5^, carbon black tinted contact lenses to reduce photophobia^27^, gold nanoparticles-loaded contact lenses to remove cystine crystals^28^, or contact lenses loaded with cysteamine^29^. However, all these are life-long treatments, and only aim at treating symptoms.

This study developed novel fibrin hydrogels, that can potentially be used for the treatment of ocular cystinosis. In the first part, biomaterials fabrication parameters were optimised using a DOE approach, whereas, in the second part, cells from a patient with cystinosis were seeded on the biomaterials.

With respect to the first part of the study, few studies have developed fibrin hydrogels for gene therapy^14–18^. However, they only compared the efficacy of two or three different fabrication systems and simply varied one-factor-at-a-time. This approach is in fact ineffective, due to the number of variable permutations (which would make it impossible to analyse all the possible combinations) and the likelihood of potential interactions between different factors.

As a matter of fact, fabrication parameters differently regulate a plethora of processes, such as DNA diffusion and release, biomaterial stiffness and degradation, cellular ability to infiltrate, migrate and proliferate. All these processes in turn affect DNA uptake and transgene expression^8^. For instance, in vivo biomaterials are required to degrade as rapidly as possible, for safety/cytotoxicity concerns. Fibrin hydrogels with low concentrations of aprotinin and thrombin are softer and degrade faster^30,31^. However, soft biomaterials generally do not transfect cells, as efficiently as stiff ones^32–35^. Furthermore, low thrombin concentrations leads to less branched networks^36–38^: this, in turn, affects cellular migration and DNA uptake^18^. Similarly, high DNA concentrations may enhance transfection. However, DNA has a high tendency to aggregate: this, in turn, may cause cytotoxicity^39^. The high number of parameters involved, and complexity of their interaction make it difficult to understand the mechanics of cellular transfection and to develop robust predictable systems, which are a basic requirement for clinical implementation. DOE is an engineering approach that allows to test several variables at the same time, with a reduced number of experiments. DOE also takes into consideration potential interactions between factors and allows the creation of predictive models^40^. DOE is only recently working its way into the biomaterial science, with a limited amount of research available. DOE was recently used to optimise cell seeding efficiency on dermal scaffolds^41^, to develop novel niosomes entrapping pregabalin^42^, to model exosome uptake^43^, to predict osteogenic differentiation of bone marrow^44^ and of adipose-derived mesenchymal stem cells^45^. This study used DOE to identify optimal concentrations of DNA, thrombin and aprotinin and, for the first time, showed the utility of this approach to the development of gene delivery constructs.

In the second part of the study, primary fibroblasts from a patient with cystinosis were seeded on the biomaterials. Cells effectively expressed the recombinant CTNS and showed a decrease in cystine content. This study also showed that adult diseased cells, if transiently transfected with CTNS, secrete the functional recombinant enzyme. As a matter of fact, it is well-known that soluble lysosomal proteins follow secretory pathways. Once in the extracellular space, they are taken up and targeted to lysosomes of deficient cells (i.e., cross-correction). However, lysosomal membrane proteins cannot be exocytosed and were traditionally considered non-secretory proteins. It was only few years ago when a few studies first observed that healthy stem cells did secrete functional CTNS^46–48^. Secretion was attributed to either extracellular vesicles^49^ or tunnelling nanotubes^50^. Although future studies are needed to identify and isolate the extracellular vesicles responsible for CTNS secretion (i.e. microvesicles or extracellular vesicles), results of this study are important in the context of gene therapy. For many tissues, indeed, transfection of the totality of resident cells is unlikely to be achieved, due to particular size, shape and anatomical localisation. Yet, phenotypic reversion of the disease could still be achieved, if a small subset of transfected cells secretes the functional enzyme.

To conclude, this study described a methodology to develop gene delivery construct by using a DOE approach, and ultimately provided new insights into the treatment of cystinosis.

## Conclusions

This study developed a fibrin-based biomaterial for controlled, localised gene delivery for the treatment of the rare disease cystinosis. Biomaterial fabrication parameters (i.e., DNA, thrombin and aprotinin concentrations) were initially optimised using the engineering approach DOE. Cells from a patient with cystinosis were then seeded on the biomaterials. Seeded cells effectively expressed the recombinant CTNS, showing a decrease in cystine content. Furthermore, conditioned media contained functional copies of the recombinant protein, that were taken up by monolayer cultures of non-transfected diseased fibroblasts. This study described a methodology to develop gene delivery constructs, by using the engineering approach DOE and ultimately provided new insights into the treatment of cystinosis.

## Supplementary Information

Supplementary Information.
