# Supplementary material for: Development of a fibrin-mediated gene delivery system for the treatment of cystinosis via design of experiment
Source: Sci Rep. 2022 Mar 8;12:3752. doi: 10.1038/s41598-022-07750-y (PMC8904479; doi:10.1038/s41598-022-07750-y)
Supplement: Supplementary file 1 — Supplementary Information. [file 41598_2022_7750_MOESM1_ESM.docx]

**Supplementary Figures**
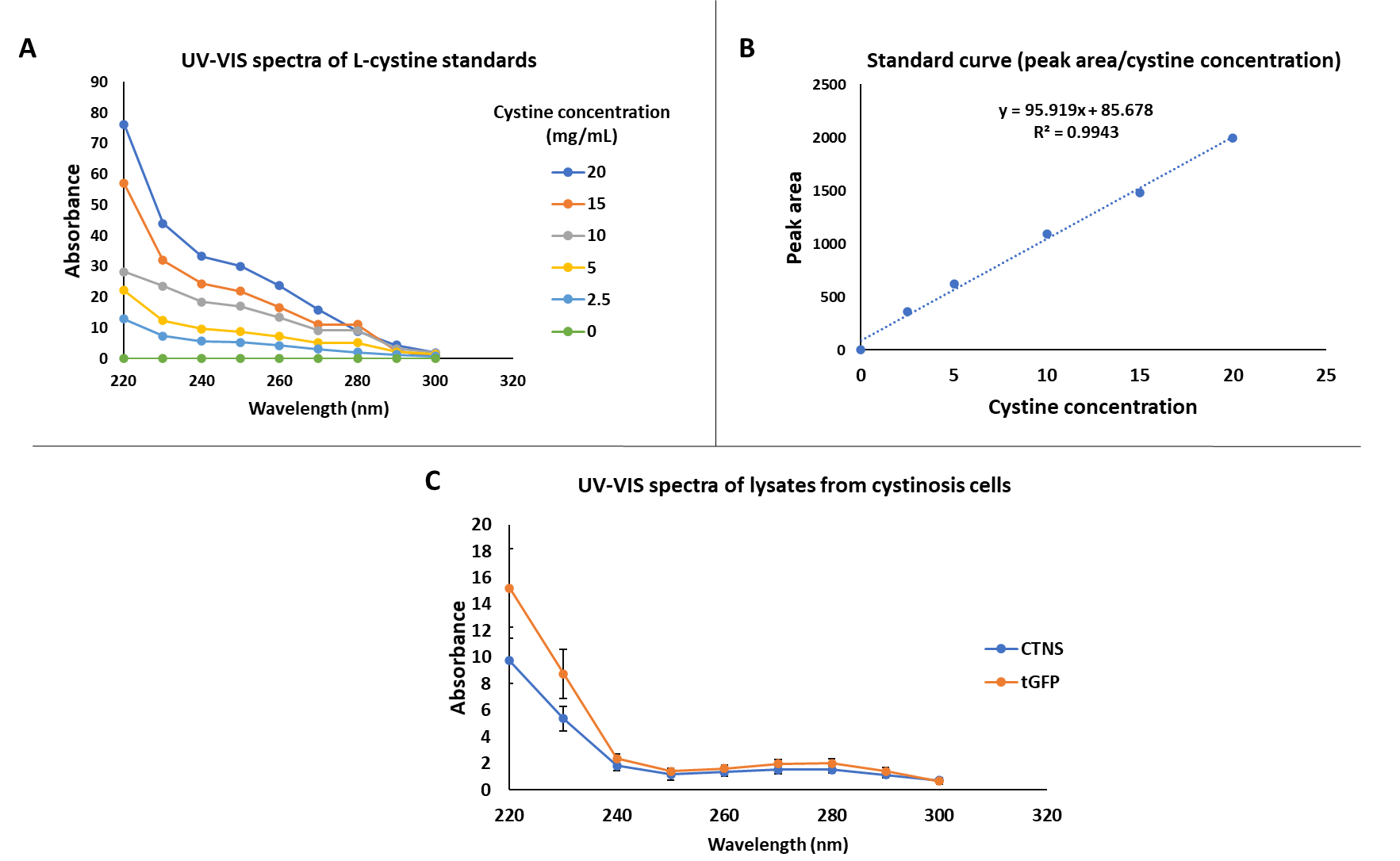


**Supplementary Figure S1: Cystine quantification through UV-VIS spectroscopy.** Five L-Cystine standards were prepared. Their absorbance at different wavelength was measured (A) and a standard curve (peak area against cystine concentration) was generated. (B) UV-VIS spectroscopy of lysates from cystinosis cells seeded on fibrin hydrogels loaded with either pCMV6-AC-CTNS-GFP (CTNS) or with pCMV6-AC-GFP (tGFP) was then generated (C). N=4


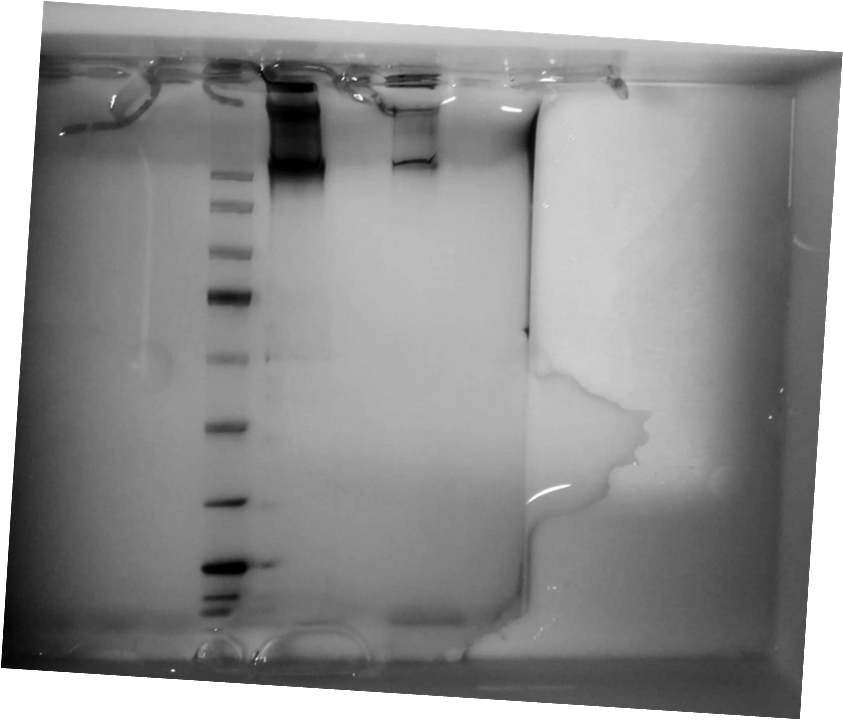


**Supplementary Figure S2:** Original uncropped gel for Figure 9C
